# The impact of the COVID-19 pandemic on academic performance: a comparative analysis of face-to face and online assessment

**DOI:** 10.3389/fpsyg.2023.1299136

**Published:** 2024-01-09

**Authors:** Carmelo Mario Vicario, Massimo Mucciardi, Pietro Perconti, Chiara Lucifora, Michael A. Nitsche, Alessio Avenanti

**Affiliations:** ^1^Department of Cognitive Sciences, Psychology, Education and Cultural Studies, University of Messina, Messina, Italy; ^2^Department of Philosophy and Communication, University of Bologna, Bologna, Italy; ^3^Department of Psychology and Neurosciences, Leibniz Research Centre for Working Environment and Human Factors at TU Dortmund, Dortmund, Germany; ^4^Bielefeld University, University Hospital OWL, Protestant Hospital of Bethel Foundation, University Clinic of Psychiatry and Psychotherapy, University Clinic of Child and Adolescent Psychiatry and Psychotherapy, Bielefeld, Germany; ^5^Dipartimento di Psicologia “Renzo Canestrari”, Alma Mater Studiorum Università di Bologna, Cesena, Italy; ^6^Neuropsychology and Cognitive Neuroscience Research Center (CINPSI Neurocog), Universidad Católica del Maule, Talca, Chile

**Keywords:** academic assessment, COVID-19 pandemic, online assessment, face-to-face assessment, archival study, archive statistical analysis

## Abstract

**Introduction:**

Survey studies yield mixed results on the influence of the COVID-19 pandemic on academic performance, with limited direct evidence available.

**Methodology:**

Using the academic platform from the Italian university system, a large-scale archival study involving 30,731 students and 829 examiners encompassing a total of 246,416 exams (oral tests only) to scrutinize the influence of the COVID-19 pandemic on the likelihood of passing exams was conducted. Examination data were collected both in face-to-face and online formats during the pandemic. In the pre-pandemic period, only face-to-face data were accessible.

**Results:**

In face-to-face examination, we observed a lower probability of passing exams during the pandemic as opposed to pre-pandemic periods. Notably, during the pandemic we found an increased chance of passing exams conducted through online platforms compared to face-to-face assessments.

**Discussion and conclusions:**

These findings provide the first direct evidence of an adverse impact of the COVID-19 pandemic on academic performance. Furthermore, the results align with prior survey studies underscoring that using telematics platforms to evaluate students' performance increases the probability of exam success. This research significantly contributes to ongoing efforts aimed to comprehend how lockdowns and the widespread use of online platforms impact academic assessment processes.

## Introduction

The COVID-19 pandemic has forced nations to undergo significant restructuring across economic, health and educational systems. Recent psychological research, spanning the past 3 years, has started to illuminate the impact of prolonged exposure to a pandemic along with associated lockdowns and home confinement on cognitive and affective processing (e.g., Diotaiuti et al., 2021, 2023; Fiorenzato et al., 2021; Wilke et al., 2021; Gewalt et al., 2022; Rania et al., 2022). For example, Fiorenzato et al. (2021), documented an increase in the severity and prevalence of conditions such as depression, anxiety disorders, abnormal sleep, appetite changes, decreased libido, and health-related anxiety in the pandemic. On the cognitive level, the authors reported a paradoxical improvement in memory, compared to pre-lockdown. However, the authors of this study reported subjective complaints of the participants with respect to daily activities involving attention, temporal orientation, and executive functions. This highlights that the effects of the pandemic on mental processes extend to both affective and cognitive dimensions.

The spread of the COVID-19 pandemic has exposed education systems to unprecedented challenges, with a sudden shift of classroom-based pedagogics to distant learning approaches (Aldossari and Chaudhry, 2021). This transition from face-to-face to virtual classes has resulted in a diverse spectrum of educational models: on the one hand, some professors replicated their in-person classes through videoconferencing, while, on the other hand, others undertook a comprehensive overhaul of their teaching plans to align methodological and evaluative strategies with the demands of the new context (Fardoun et al., 2020; Ramos-Pla et al., 2021). For instance, there's an observable trend of increasing collaborative work (Ramos-Pla et al., 2022), which enhances professor-student interactions—a critical predictor of students' perceived quality of teaching (del Arco et al., 2021). In response to this paradigm shift, training centers across various universities adapted their programs to facilitate the continuous learning of professors. However, these educators faced challenges, expressing concerns about the time constraints in assimilating new knowledge into their teaching practices and the complexities of online evaluations (Ramos-Pla et al., 2021). Moreover, other studies underscored students' difficulties in following online courses, particularly those without personal devices or sharing them with other family members (Ramos-Pla et al., 2023).

In the present study, we focused on academic assessment, a pivotal sector significantly impacted by the pandemic (Onyema et al., 2020; Rashid and Yadav, 2020; Estrada Guillén et al., 2022; Gewalt et al., 2022). This sector witnessed an extensive adoption of telematic technologies and was a dynamic response to ensure the continuity of educational services, including university services.

To the best of our knowledge, the existing literature (e.g., Mahdy, 2020; Radu et al., 2020; Son et al., 2020; Akin-Odanye et al., 2021; Andersen et al., 2022; Appleby et al., 2022; Hadwin et al., 2022) exploring the impact of the COVID-19 pandemic on academic performance is based on conventional survey research methodology. For instance, Mahdy (2020) examined the academic performance of veterinary medical students during the pandemic by collecting their opinions via an online Google form questionnaire. The author pointed out that while online education offers an opportunity for self-study, the main pandemic-related challenge in veterinary medical science is how to give practical lessons. Moreover, the study by Estrada Guillén et al. (2022) identified a connection between emotional intelligence and resilience to pandemics, which was associated with better academic performance. This can help to explain the mixed results provided by the literature in the field (e.g., Gonzalez et al., 2020; Giusti et al., 2021; Keržič et al., 2021).

Traditional–internet-based survey panels are characterized by several limitations such as response or sampling biases, desirability biases, and memory recall biases (Andrade, 2020). Moreover, the sampled data might not be representative of the actual population (Hays et al., 2015), potentially yielding biased results.

In the current study, we aimed to overcome such limitations by examining actual data recorded and archived within our university multifunction academic (online) platform to answer a series of outstanding questions not addressable via survey studies. This platform serves as a comprehensive teaching management computer system, providing students and professors with a dedicated space to oversee exam registration, grade management, and participation in university initiatives. The wealth of information available through this platform includes details about the scheduling of all exams, and the outcomes of each student evaluated within our university. This dataset thus provides a more reliable and accurate picture of the impact of the COVID-19 pandemic on academic achievement compared to survey studies. Furthermore, these actual data serve as a robust alternative to subjective survey measures, which are susceptible to biases, including those stemming from social expectations. Finally, this dataset allowed us to explore whether and how the mode of examination (face-to-face vs. online platform) during the pandemic influences its impact.

Our focus was directed to data spanning the period between January 2019 and October 2021. This specific time frame facilitated a comparative analysis, allowing us to discern any differences between “in-person” and “online” examinations, both in the period just before and during the pandemic. Additional details are offered in the Methods section.

## Methods

The data were extracted from the multifunction academic platform of the University of Messina. These data consisted of 246,416 assessments (exams) provided by 829 examiners. The evaluation involved 1,846 teaching courses. The data were collected over three academic years, from 2019 to 2021, and involved a total of 32,123 students [originating from 135 bachelor's/master's degrees and post-graduate specializations offered by the University of Messina (see Table 1)].

**Table 1:** Number of assessments and respective percentages per type of degree course before and during the COVID-19 pandemic.

**Type of the degree course**	**Presence (no pandemic)**		**Presence (pandemic)**		**Online (pandemic)**		**Total**	
	**Count**	**%**	**Count**	**%**	**Count**	**%**	**Count**	**%**
Degree course (3 years)	63,420	60.60	27,802	59.13	53,112	56.06	144,334	58.57
Master's degree (2 years)	15,016	14.35	7,406	15.75	13,183	13.92	35,605	14.45
Master's degree (5 years single-cycle)	24,160	23.08	5,828	12.39	13,315	14.05	43,303	17.57
Master's degree (6 years single-cycle)	1,987	1.90	5,691	12.10	9,837	10.38	17,515	7.11
Teaching specialization (after master's degree)	0	0.00	0	0.00	5,161	5.45	5,161	2.09
Medical specialization (after master's degree)	75	0.07	295	0.63	128	0.14	498	0.20
Total	104,658	100.00	47,022	100.00	94,736	100.00	246,416	100.00

The pre-pandemic period refers to exams from January 2019 to February 2020. The pandemic period refers to exams from March 2020 to October 2021. We choose to include a relatively extended time window for the pandemic condition as two modalities of examination (i.e., in presence and online) were implemented in this period. In contrast, only one (in person) was available in the pre-pandemic condition. We excluded data referring to mixed mode (online/in person) assessments, as it was not possible to disentangle the two modalities. Refer to Tables 2, 3 for more details.

**Table 2:** Comparison between complete data and selection by exam type.

		**Exams**		**Total**
		**Not selected**	**Selected**	
Exam type	Written and oral	4,590	0	4,590
	Written	20,927	0	20,927
	Oral	63,542	246,416	309,958
Total		89,059	246,416	335,475

**Table 3:** Comparison between complete data and selection by modality of the assessment.

			**Exams**		**Total**
			**Not selected**	**Selected**	
Modality of the assessment	Presence (no pandemic)	Count	20,290	104,658	124,948
		% Within modality of the assessment	16.2%	83.8%	100.0%
	Presence (pandemic)	Count	6,488	47,022	53,510
		% Within modality of the assessment	12.1%	87.9%	100.0%
	Online (pandemic)	Count	1,443	94,736	96,179
		% Within modality of the assessment	1.5%	98.5%	100.0%
Total		Count	28,221	246,416	274,637
		% Within modality of the assessment	10.3%	89.7%	100.0%

The extracted data included the modality of the assessment session (online and in presence), the type of assessment (written and oral), and the respective outcome (passed or failed). Inclusion criteria for the final data analysis referred to only oral examinations. We excluded data referring to mixed mode (online/presence) assessments, as it was not possible to clearly disentangle the two modalities. We referred to rectoral decrees to determine when the exams were (or not) online or in the mixed mode. For privacy reasons, demographic data (e.g., age, sex, and country of origin) were not provided. The study was approved by the Local Ethics Committee (Protocol Number: COSPECS_08_2022). The ethics committee waived the requirement for consent as the study implied the analysis of already collected and anonymized data.

A typical oral exam session begins with verifying the student's identity. There is no standard way to conduct the exam. The assessor can start the session by asking the student to choose the topic from the general program of the course or by selecting the topic himself from those addressed in the course. The duration of the exam and the number of questions also vary depending on the assessor and the need to have a clear picture of the level of preparation of the student being examined. For data analysis we employed an approach to discern significant differences in pass rates between categories. Specifically, we utilized the prop.test() function in the R language, which conducts a hypothesis test to compare proportions. Internally, this function employs the chi-square test statistic for proportions. The version used for this analysis is R language ver. 4.2.

## Results

First, the overall number of assessments during the pandemic was higher (*N* = 141.758) compared to the pre-pandemic period (*N* = 104.658). However, when looking separately at each type of degree course (Table 1), a reversed pattern of results (i.e., a lower number of assessments) is documented for the master's degree (5 years single cycle).

Table 4 provides the results of the statistical analysis when comparing the number of assessments before vs. during the pandemic for each type of degree course.

**Table 4:** Statistical comparisons of the number of assessments as a function of assessment modalities (A = in presence before the pandemic; B = in presence during the pandemic; C = online during the pandemic) for each type of degree course.

**Type of the degree course**	**Modality of the assessment**		
**Presence (no pandemic)**	**Presence (pandemic)**	**Online (pandemic)**
**(A)**	**(B)**	**(C)**
Degree course (3 years)	B, C	C	
Mas'er's degree (2 years)	C	A, C	
Mas'er's degree (5 years single-cycle)	B, C		B
Mas'er's degree (6 years single-cycle)		A, C	A
Teaching specialization (after mas'er's degree)	^a^	^a^	
Medical specialization (after mas'er's degree)		A, C	A

Letters in each cell indicate significant pairwise differences between assessment modalities (actual column vs. the other ones) based on two-sided z-tests using the Bonferroni correction.

a This category is not used for comparisons because its column proportion is equal to zero.

**Figure 1:**
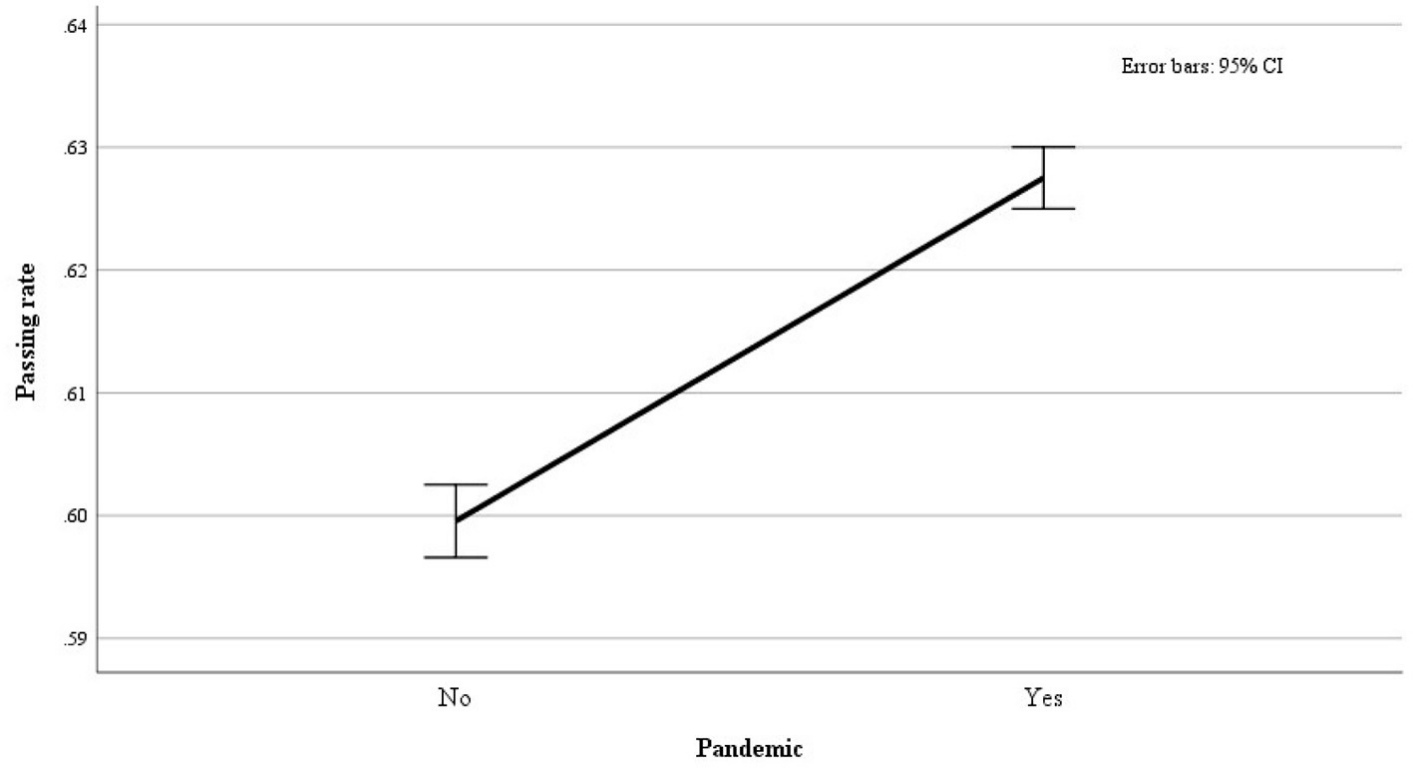
The figure shows the pass rates in the COVID-19 pandemic (Yes) and before (No).

**Figure 2:**
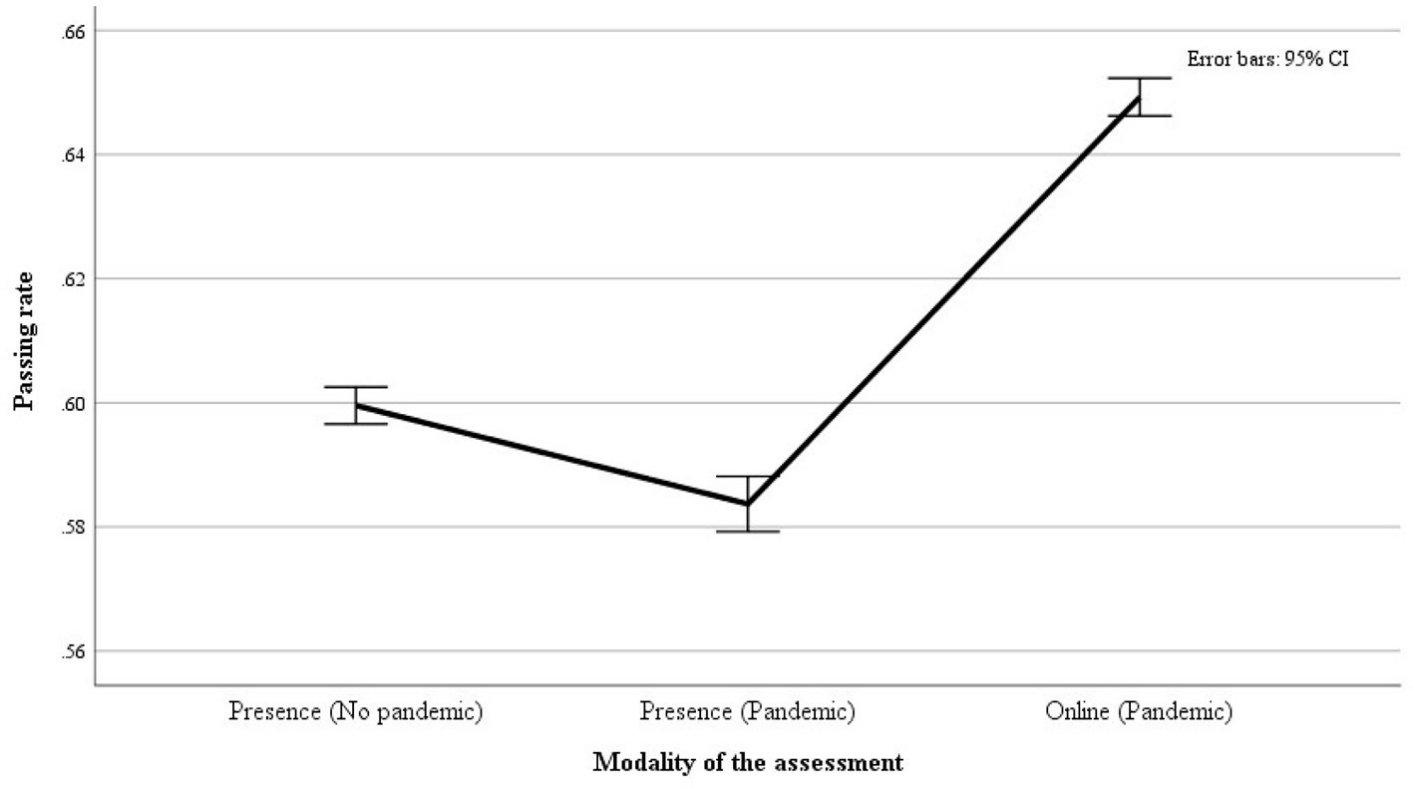
The figure shows the pass rate by modality of the assessment.

Considering the whole sample, the observed absolute number of passed exams was 151.702 out of 246.416, resulting in an overall pass rate of 0.62 (61.6%). Furthermore, we noted an overall higher chance of a favorable assessment during the COVID-19 pandemic compared to the pre-pandemic period (0.63 vs. 0.60) (Figure 1 and Table 5). However, a more mixed picture emerged when examining different assessment modalities. Specifically, during the COVID-19 pandemic, the chance for a favorable assessment was higher in the “online” modality (0.65) but notably lower in the “in presence” modality (0.58), compared to the pre-pandemic “in presence” assessments (0.60). This indicates that the pandemic negatively influenced academic performance, specifically when the assessment was conducted in the standard (i.e., in presence) setting. See Table 6 and Figure 2.

**Table 5:** Pass rate by the assessment modality: number of examinations, standard error (S.E.), and *Z-*test for equality of proportions.

**Pandemic**	**Pass rate**	**Exams**	**S.E**.
No (before February 2020)	0.60	104.658	0.002
Yes (after February 2020)	0.63	141.758	0.001
Total (full temporal range)	0.62	246.416	0.001
*Z-*test = 14.1 *p* < 0.0001			

**Table 6:** Pass rate by modality of the assessment: number of examinations, the respective standard error (S.E.), and *Z-*test.

**Modality of the assessment**	**Pass rate**	**Exams**	**S.E**.
Presence (no pandemic)	0.60	104,658	0.002
Presence (pandemic)	0.58	47,022	0.002
Online (pandemic)	0.65	94,736	0.002
Total	0.62	246,416	0.001
***Z-*** **test**	**Sig**.
Presence (no pandemic) vs. presence (pandemic)		5.8	*p* < 0.001
Presence (no pandemic) vs. online (pandemic)		22.9	*p* < 0.001
Presence (pandemic) vs. online (pandemic)		24.0	*p* < 0.001

To account for potential season-related effects, we performed a further control analysis comparing passing rates between three consecutive years (2019, 2020, 2021) considering the same seasons for each of the 3 years. We excluded the winter season from the analysis because it encompassed both pre-pandemic and pandemic data, in accordance with the university's rectoral decree, which established the examination modality (online, face-to-face) for the entire institution. We examined the influence of seasons, and assessment modality on the likelihood/chance of a favorable assessment.

The results (two-sided *Z*-tests) confirm the pattern observed in the primary analysis, indicating a reduced likelihood of a favorable assessment in face-to-face settings during the pandemic (58%, *p* = 0.007), and an increased likelihood of a favorable evaluation online (during the pandemic, 68%, *p* < 0.001) compared to the pre-pandemic—face-to-face—setting condition (59%). See Table 7 for details on the different seasons.

**Table 7:** Pass rate by modality of the assessment after controlling for the season effect.

**Modality of the assessment**		**Exams**	**Passing rate**	**95% Confidence interval**	
				**Lower bound**	**Upper bound**
Presence (no pandemic)	Spring_2019	23,910	0.62	0.62	0.63
	Summer_2019	22,138	0.58	0.57	0.58
	Autumn_2019	12,981	0.56	0.55	0.57
	Total	59,029	0.59	0.59	0.60
Presence (pandemic)	Spring_2021	11,544	0.67	0.67	0.68
	Summer_2021	30,438	0.56	0.55	0.57
	Autumn_2021	5,040	0.52	0.51	0.53
	Total	47,022	0.58	0.58	0.59
Online (pandemic)	Spring_2020	26,807	0.67	0.67	0.68
	Summer_2020	9,887	0.66	0.65	0.66
	Autumn_2020	6,899	0.54	0.53	0.56
	Spring_2021	6,395	0.88	0.88	0.89
	Total	49,988	0.68	0.67	0.68

## Discussion

In this archival study we examined the impact of the COVID-19 pandemic on academic assessment outcomes, introducing several innovative elements compared to previous work in the field. Our approach combined direct empirical evidence about academic performance, a comprehensive archival analysis of large-scale data, and a comparison between face-to face and online assessments.

The first important finding is the significant difference of exam pass rates between face-to-face and online modalities. This has relevant practical implications for the landscape of academic assessment. In contrast to previous survey-based studies (e.g., Mahdy, 2020), our research, based on direct empirical evidence, demonstrates that the COVID-19 pandemic negatively affected academic assessments, specifically in a face-to-face setting. This is evident through a decreased pass rate in the “in person” assessments during the pandemic compared to the period before the outbreak. Crucially, this trend persists even when accounting for seasonal effects, and might be caused by an adverse impact of the COVID-19 pandemic on mental health (Salehinejad et al., 2020; Craparo et al., 2022; La Rosa et al., 2022; Vicario et al., 2023) and cognitive skills (Fiorenzato et al., 2021), which could have deleterious effects on academic performance.

In principle, the lower pass rate in “face-to-face” assessments during the COVID-19 pandemic may also be influenced negatively by attendance in online classes provided during the pandemic, which could have affected learning quality. However, we observed a higher pass rate for online assessments during the pandemic (but see discussion below), and evidence from other studies suggests that online platforms and other modalities for remote practices, such as clinical interventions (D'Oliveira et al., 2022; Prato et al., 2022) and remote learning (Al-Maroof et al., 2021) allow for effective outcomes. Therefore, although we do not dismiss the possibility that online lectures may have negatively affected learning in some students, our data and previous research (Al-Maroof et al., 2021) argue against attributing a causal role to this factor. On the other hand, “face-to-face” exams might have triggered heightened social stress, originating from prolonged isolation, which restricts social interactions. This, in turn, could have impacted students' cognitive performance and assessors' decision-making processes in the assessment (e.g., Starcke and Brand, 2012).

Other potential stressors, such as using facial masks, may have further reduced pass rates by interfering with student performance. The discomfort associated with face masking (e.g., Lazzarino et al., 2020; Tornero-Aguilera and Clemente-Suárez, 2021) has been shown to compromise cognitive performance and interfere with the occupational duties of workers (e.g., Shenal et al., 2012), and prolonged mask use can cause bilateral headaches (Ong et al., 2020). Face masks may compromise the positive effects of relational continuity (Wong et al., 2013). Additionally, facial masking reduces the recognition of emotions, potentially impacting social functioning (Grundmann et al., 2021).

The second major finding in this study is the higher pass rate observed in the online assessment condition during the pandemic, suggesting a potential advantage for students tested in this modality. Also, this outcome remains consistent even after accounting for seasonal effects, indicating that the utilization of online platforms for assessment could increase the likelihood of passing exams during the pandemic. It is important to note, however, that no data on online assessments conducted before the COVID-19 pandemic are available, making it challenging to determine whether the increased pass rate is solely due to the use of the online platform or reflects an interaction between this assessment modality and the unique circumstances of the pandemic.

The more favorable outcome in the online session could potentially be attributed to a reduction in social distress experienced by students. This hypothesis is supported by the study of Stowell and Bennett (2010), indicating that students who typically experience high levels of test anxiety in a classroom setting report reduced test anxiety when taking exams online. This might reflect an effective capacity to implement successful coping strategies crucial for an effective adaptation to unexpected circumstances associated with the ongoing pandemics (e.g., Zhao et al., 2022).

However, it is noteworthy that approximately one-third of students perceive e-exams as more stressful than in-person exams (Elsalem et al., 2020).

Additionally, it is essential to acknowledge that previous research has emphasized an increased likelihood of cheating in (online) exams when lacking proctoring mechanisms (as in this case) (e.g., Harmor and Lambrinos, 2008; see also Chiang et al., 2022, for a recent systematic review of academic dishonesty in online learning environments). This underscores the potential risk of undeserved promotion associated with the use of telematic tools. However, it is important to recognize the relevance of these technologies in supporting the continuity of teaching and academic assessment during the challenging circumstances posed by the COVID-19 pandemic.

Our study significantly contributes to understanding how the COVID-19 pandemic has influenced academic assessment, shedding light on both challenges and opportunities associated with online platforms. We provide direct evidence of the adverse effects of the COVID-19 pandemic on academic performance when exams are conducted in person. Conversely, the observed higher pass rate in the online condition, compared to the in-person conditions both before and during the pandemic, suggests a potential drawback of this assessment modality. This includes an increased likelihood for students to consult notes and teaching material in the absence of a supervision system, and/or a higher inclination of assessors toward positive evaluations. However, it is important to note that this statement, which represents the main limitation of our work, remains unverified, as our study did not encompass the condition of online assessment before the COVID-19 pandemic for a comparative analysis with that during the pandemic. Moreover, other limitations pertain to the absence of control for additional variables that could have influenced the results, such as variations in learning styles, assessment methodologies, and socio-cultural factors.

## Conclusions

In conclusion, our study extends the existing body of research (e.g., del Arco et al., 2021; Diotaiuti et al., 2021; Ramos-Pla et al., 2021, 2022), underscoring the profound impact of the COVID-19 pandemic on academic assessments and the use of virtual classes. For face-to-face examinations, it documents a lower probability of passing an exam during the pandemic compared to pre-pandemic times. It also emphasizes disparities in pass rates between in-person and online assessments, indicating a higher likelihood of passing exams online compared to in-person. Potential factors that contribute to explaining these differences include the impact of the pandemic on students' mental wellbeing and/or the potential for academic dishonesty in online assessments. The discovery of a lower probability of passing exams during the pandemic compared to pre-pandemic times suggests educational institutions need to formulate resilient contingency plans, crucial for mitigating disruptions in academic assessments resulting from unforeseen events such as pandemics.

The finding that the use of online platforms for assessment may increase the likelihood of passing exams holds practical implications for assessment strategies. It unveils, among other considerations, the potential risk of overestimating student‘s knowledge of the subject matter, which needs to be addressed.

## Data availability statement

The raw data supporting the conclusion of this article will be available by sending a formal request to COSPECS Department at dip.cospecs@pec.unime.it.

## Ethics statement

The studies involving humans were approved by Local Ethics Committee, Cospecs Department, University of Messina. The studies were conducted in accordance with the local legislation and institutional requirements. Written informed consent for participation was not required from the participants or the participants' legal guardians/next of kin because we used archival data to conduct our study.

## Author contributions

CV: Conceptualization, Investigation, Methodology, Resources, Validation, Visualization, Writing – original draft, Writing – review & editing. MM: Conceptualization, Data curation, Formal analysis, Investigation, Methodology, Resources, Software, Supervision, Validation, Visualization, Writing – review & editing. PP: Conceptualization, Resources, Supervision, Visualization, Writing – review & editing. CL: Conceptualization, Writing – review & editing. MN: Methodology, Supervision, Validation, Writing – review & editing. AA: Conceptualization, Supervision, Validation, Writing – original draft, Writing – review & editing.
